# The Root Herbivore History of the Soil Affects the Productivity of a Grassland Plant Community and Determines Plant Response to New Root Herbivore Attack

**DOI:** 10.1371/journal.pone.0056524

**Published:** 2013-02-18

**Authors:** Ilja Sonnemann, Stefan Hempel, Maria Beutel, Nicola Hanauer, Stefan Reidinger, Susanne Wurst

**Affiliations:** 1 Freie Universitaet Berlin, Dahlem Centre of Plant Sciences, Berlin, Germany; 2 University of York, Department of Biology, York, United Kingdom; University of Leipzig, Germany

## Abstract

Insect root herbivores can alter plant community structure by affecting the competitive ability of single plants. However, their effects can be modified by the soil environment. Root herbivory itself may induce changes in the soil biota community, and it has recently been shown that these changes can affect plant growth in a subsequent season or plant generation. However, so far it is not known whether these root herbivore history effects (i) are detectable at the plant community level and/or (ii) also determine plant species and plant community responses to new root herbivore attack. The present greenhouse study determined root herbivore history effects of click beetle larvae (Elateridae, Coleoptera, genus *Agriotes*) in a model grassland plant community consisting of six common species (*Achillea millefolium*, *Plantago lanceolata*, *Taraxacum officinale*, *Holcus lanatus*, *Poa pratensis*, *Trifolium repens*). Root herbivore history effects were generated in a first phase of the experiment by growing the plant community in soil with or without *Agriotes* larvae, and investigated in a second phase by growing it again in the soils that were either *Agriotes* trained or not. The root herbivore history of the soil affected plant community productivity (but not composition), with communities growing in root herbivore trained soil producing more biomass than those growing in untrained soil. Additionally, it influenced the response of certain plant species to new root herbivore attack. Effects may partly be explained by herbivore-induced shifts in the community of arbuscular mycorrhizal fungi. The root herbivore history of the soil proved to be a stronger driver of plant growth on the community level than an actual root herbivore attack which did not affect plant community parameters. History effects have to be taken into account when predicting the impact of root herbivores on grasslands.

## Introduction

Insect root herbivores can alter plant community structure by affecting the competitive ability of single plants [1]. However, their effects can be modified by the soil environment [2]–[3]. Root herbivory itself may induce changes in the soil biota community [4]–[6]. It has recently been shown that these changes can affect plant growth in a subsequent season or plant generation [7]. However, it is not known whether these root herbivore history effects (i) are detectable at the plant community level and/or (ii) also determine plant species and plant community responses to new root herbivore attack.

Several studies documented that root symbiotic arbuscular mycorrhizal fungi (AMF) and root herbivores influence each other's performance and effect on the host plant [8]–[11]. One study [12] even found consequences of these interactive effects on the plant community level. AMF provide mineral nutrients to the plant in exchange for photosynthates [13]. Their effect on plant growth proved to be plant as well as AMF species specific [14]. As root herbivores can alter AMF community structure [15] their history effects may potentially be generated through changes in this important soil biota group.

Click beetle larvae (Elateridae, Coleoptera) of the genus *Agriotes* are dominant generalist root herbivores in European grasslands [16]–[18] and also pests in different economically important crops. While their effects on cropping systems are well studied, their impact on grassland plant communities is almost unknown. Studies that included measurements on background soil biota have so far only been done in a single plant system (*Plantago lanceolata*), and found no effects of *Agriotes* larvae on the microbial carbon source utilization in the rhizosphere [19] and root colonization by arbuscular mycorrhizal fungi [20]–[21].

The presented greenhouse study aimed at determining root herbivore history effects of *Agriotes* spp. larvae on a grassland plant community. The root herbivore history of the soil was generated by growing a model plant community without or with *Agriotes* larvae in soil biota communities from two different grassland sites in a first phase of the experiment. Root herbivore history effects on plant growth and response to a new *Agriotes* attack were determined by growing the model plant community without or with *Agriotes* larvae in the either herbivore untrained (absence of *Agriotes* larvae in phase 1) or trained (presence of *Agriotes* larvae in phase 1) soil, in a second phase of the experiment. We further investigated effects of present or past *Agriotes* herbivory on AMF as one important group within the soil biota community. Measurements included AMF community parameters in soil as well as community parameters and colonization levels in the roots of the model plant *P. lanceolata.* We hypothesized that (i) the root herbivore history of the soil influences plant growth and response to a new root herbivore attack, (ii) this has consequences for plant community structure, and (iii) root herbivore history effects can be explained by herbivore induced shifts in the AMF community.

## Materials and Methods

The experiment was conducted within the frame of the Biodiversity Exploratories project [22]. Background soil and inocula were collected from grassland sites in the Schorfheide exploratory, 100 km north of Berlin, Germany. A general field work permit was issued by the Landesumweltamt Brandenburg. The collection did not involve endangered or protected species.

### Background soil

The background soil was an alfisol that was collected from the upper 5–30 cm of a mown pasture. Stones and coarse roots were sieved out (1 cm mesh size) and the soil was steamed for 4 h at 90°C prior to usage to kill root herbivores.

### Establishment of plant community and *Agriotes* treatment

A total of 120 round 2 L plastic pots (Albert Treppens & Co Samen GmbH, Berlin, Germany) were prepared by sealing the drainage holes with water permeable non-woven material (Plantex®, DuPont, Germany) to prevent escape of *Agriotes* larvae in *Agriotes* treatments. Pots were filled with background soil, and soil biota other than soil macrofauna was reintroduced by means of inocula that were specific for the two phases of the experiment (see detailed experimental set up of phase 1 and 2 below). A plant community consisting of six grassland plant species (*Achillea millefolium* L., *P. lanceolata* L., *Taraxacum officinale* Wiggers, *Holcus lanatus* L., *Poa pratensis* L., *Trifolium repens* L.) was established in each pot, with one individual per species. The plant species chosen were common on the two grassland sites from where the soil biota inocula for phase 1 (see below) were collected. The proportion of plant functional types (two grasses, three herbs and one legume) resembled those at the sites. Pots in each treatment were allocated to three different sowing schemes to account for neighboring effects, and plant species were sown at evenly distanced positions in each pot, with four seeds (Appels Wilde Samen GmbH, Germany) per position, according to the sowing schemes. Each pot was covered with a perforated plastic bag (15×66 cm, EDNA International GmbH, Germany) to prevent invasion of unwanted herbivores. Seedlings were thinned to one per position approximately one week after sowing. Pots were randomized once a week and watered from the bottom as needed during the course of the experiment. To establish the *Agriotes* treatment, *Agriotes* spp. larvae, collected from a fallow grassland app. 10 km south of Berlin, were added to half of the pots in each soil biota/training treatment (see below) four weeks after sowing, with three larvae per pot. Five randomly chosen larvae from that fallow grassland were identified as *A. obscurus* [23]. The composition of instars reflected those at the site but was not assessed in detail. Larvae were randomly allocated to the pots. Larvae were rinsed with tap water prior to addition. The rinsing water was collected and evenly allocated to *Agriotes*-free pots to correct for microorganisms that were introduced with the larvae.

### Experimental set up

#### Phase 1

40 pots were each filled with 1790+/−1 g of background soil. The upper 790 g of background soil in each pot were mixed with 174+/−1 g of one of two soil biota inocula, with 20 pots per inoculum. Each inoculum consisted of un-steamed soil that had been collected from the upper 10 cm of one of two alfisol grassland sites (SEG 33 and SEG 37). Sites for inocula collection were chosen (i) to be of the same soil type as the background soil to ensure establishment of the soil biota in the pots and (ii) to differ in management (SEG 33: fertilized mown pasture, SEG 37: unfertilized pasture) to maximize differences between the two soil biota communities in general and AMF communities [24] in particular. Macrofauna and coarse material was sieved out (4 mm mesh size) from the inocula, roots were cut to 1 cm pieces and added back, and the inocula were air dried prior to usage to kill remaining root herbivore eggs. The plant community and *Agriotes* treatment were established as described above, resulting in four treatments (two soil biota communities (SEG 33, SEG 37), either without or with *Agriotes* larvae), with 10 replicates each. Plants were grown for two months in a climate chamber at 20/18°C day/night temperature, 69% air humidity and 16 h day length.

#### Phase 2

80 pots were each filled with 1140+/−1 g of background soil. The upper 540 g of background soil in each pot were mixed with 825+/−1 g of a soil biota inoculum. Soil biota inocula consisted of root free soil from phase 1 pots. The comparably high amount of inoculum was chosen to transfer not only the soil biota themselves, but also the respective abiotic conditions that potentially resulted from the treatments in phase 1. After the harvest of phase 1, *Agriotes* larvae were removed from the respective treatments and the soil of each phase 1 pot was well mixed and air dried for storage until the set up of phase 2. The soil from one phase 1 pot then served as inoculum for two pots in phase 2. The plant community and *Agriotes* treatment were established as described above, with each pair of pots being split between control and *Agriotes* treatment. This resulted in eight treatments (two soil biota communities (SEG 33, SEG 37), either untrained or *Agriotes* trained during phase 1 (together with respective abiotic soil conditions), each without or with new *Agriotes* larvae in phase 2), with 10 replicates each. Plants were grown for two months in June/July in a greenhouse at 20/19°C minimal day/night temperature with 16 h additional light per day.

### Harvests and measurements

Each phase was harvested by cutting the shoots at soil surface level. Shoot biomass for each plant was determined gravimetrically after drying at 56°C for 72 h as gram dry weight (gDW). Shoot biomass data (means (se)) for all plant species grown in different treatments are reported as supporting information (Supporting Tables S1). Shannon's diversity index was calculated per pot as H′ = ∑pi*ln pi, where pi is the share of shoot biomass represented by species i and is calculated as shoot biomass i/shoot biomass total. Roots were sieved (1 mm mesh size) from the soil, and *P. lanceolata* roots were separated as far as possible. As roots were strongly entangled it was not possible to further separate plant species. Thus, total roots per pot were washed, and root biomass (including *P. lanceolata*) per pot was determined gravimetrically after drying at 56°C for 72 as gDW.

Soil carbon (C) and nitrogen (N) content was determined from air dried soil of the original soil biota inocula and of phase 1 pots at harvest by means of complete combustion and chromatographical detection (analyzer EuroEA, HEKATech GmbH, Germany). Soil CN ratio was calculated as surrogate for nutrient mineralization [25]–[27].

Mycorrhizal structures in *P. lanceolata* roots from all pots in phase 1 and half of the pots in each treatment in phase 2 were stained with the ink-vinegar method [28]. The percentage of root length colonized [%RLC] by arbuscules and mycorrhizal structures in total (total AMF, including arbuscules, vesicles and intraradical hyphae) was determined at 200 fold magnification using the gridline intersect method [29]. The length of extraradical AMF hyphae in soil (LEH [m/g soil]) was determined for phase 1 and 2 from half of the pots in each treatment applying the methods of [30] and [31]. The AMF community was characterized in phase 1 and 2 from fresh substrate (stored at −80°C until analysis) and dried *P. lanceolata* roots by means of terminal restriction fragment length polymorphism (TRFLP) using the database TRFLP approach as outlined in [32]. Briefly, soil DNA was extracted from root and soil samples of each pot and amplified using AM fungal specific primers for the ribosomal small subunit gene (SSU). PCR amplicons were pooled for root and soil samples separately, cloned and 129 clones were sequenced to obtain a database of AM fungal operational taxonomic units (OTUs) present in the samples. This database was used to calibrate the T-RFLP approach, which was then applied on the PCR products obtained from each sample separately. Obtained TRFLP peaks were compared with the database to identify present OTUs using the TRAMPR package in R [33]. A detailed description of the methods is available as supporting information (Protocol S1). AMF data (means (se)) in different treatments are reported as supporting information (Supporting Tables S1).

### Statistical analyses

All statistical analyses were performed using the software program ‘R’, version 2.12.0 [34]. Data were analyzed separately for phase 1 and 2 of the experiment. Effects of sowing scheme, soil biota- and Agriotes treatments on plant biomass parameters, *P. lanceolata* root length colonized by AMF, length of extraradical AMF hyphae (LEH) and soil CN ratio were analyzed with generalized least square models (GLS) for phase 1 and linear mixed effects models (lme) for phase 2. Identities of phase 1 pots were included as random factor in the models to analyze effects in phase 2. Inhomogeneity of variances among data of different treatments was accounted for by applying the varIdent command [35]. The factor sowing scheme did not affect any of the parameters and was therefore excluded from the models. Effects of soil biota- and Agriotes treatments on AMF community composition were analyzed with permutation tests based on Jaccard distance matrices using the adonis command [36]. AMF species extracted from P. lanceolata roots in phase 1 could not be analyzed due to very low species detection. To visualize AM fungal communities in the pots of phase 2, we calculated a non-metric analysis and plotted mean community composition and standard error for the four treatment combinations of soil biota and *Agriotes* training.

## Results

The two phases of the experiment differed in plant biomass and community composition. In phase 1, total plant biomass per pot was on average 3.5 times higher than in phase 2 (Table 1). *T. repens* clearly dominated the plant community at the harvest of phase 1, while in phase 2 the plant community was only slightly dominated by *P. lanceolata*.

**Table 1:** Plant and AMF parameters and Soil CN ratio (mean (se)) in different soil treatments (soil biota communities SGE 33 and SEG 37, *Agriotes* untrained (−AP1) or trained (+AP1) soil substrate) in phase 1 and 2.

	Phase 1		Phase 2			
	SEG 33	SEG 37	SEG 33	SEG 33	SEG 37	SEG 37
			(−AP1)	(+AP1)	(−AP1)	(+AP1)
Shannon's H	1.29 (0.04)	1.27 (0.03)	1.53 (0.03)	1.51 (0.03)	1.39 (0.03)	1.40 (0.04)
Plant biomass total (gDW)	20.73 (0.83)	22.18 (0.92)	5.70 (0.33)	6.32 (0.31)	6.13 (0.30)	6.54 (0.18)
Root biomass total (gDW)	7.72 (0.43)	9.23 (0.42)	2.01 (0.17)	2.33 (0.15)	2.38 (0.22)	2.56 (0.16)
Shoot biomass total (gDW)	13.00 (0.59)	12.95 (0.75)	3.70 (0.17)	3.99 (0.17)	3.74 (0.15)	3.99 (0.14)
Shoot biomass (gDW)						
*A. millefolium*	1.21 (0.16)	0.66 (0.14)	0.42 (0.06)	0.46 (0.05)	0.67 (0.12)	0.47 (0.05)
*P. lanceolata*	1.82 (0.21)	1.91 (0.27)	0.97 (0.10)	1.10 (0.08)	1.25 (0.14)	1.16 (0.11)
*T. officinale*	0.25 (0.04)	0.35 (0.07)	0.75 (0.11)	0.81 (0.12)	0.76 (0.14)	1.19 (0.14)
*H. lanatus*	3.01 (0.24)	3.85 (0.29)	0.91 (0.15)	1.10 (0.18)	0.63 (0.15)	0.93 (0.16)
*P. pratensis*	0.23 (0.03)	0.25 (0.04)	0.07 (0.02)	0.09 (0.01)	0.02 (0.01)	0.02 (0.01)
*T. repens*	6.49 (0.74)	5.94 (0.71)	0.57 (0.10)	0.46 (0.08)	0.42 (0.11)	0.22 (0.05)
LEH [m/gDW soil]	0.47 (0.07)	1.24 (0.21)	2.07 (0.47)	1.16 (0.29)	1.40 (0.25)	1.87 (0.42)
No of AMF species in soil	3.75 (0.61)	5.15 (0.23)	2.40 (0.26)	3.33 (0.52)	3.50 (0.49)	2.61 (0.52)
No of AMF species in roots			3.00 (0.48)	2.90 (0.43)	4.25 (0.45)	3.83 (0.46)
Total AMF [% *P. lanceolata* RLC]	18.95 (2.37)	24.05 (2.50)	71.50 (3.00)	66.80 (3.81)	59.80 (3.53)	46.50 (3.72)
Arbuscules [% *P. lanceolata* RLC]	8.20 (1.81)	5.70 (0.95)	16.50 (2.66)	17.10 (2.99)	12.50 (1.60)	10.75 (2.12)
Soil CN ratio	12.09 (0.15)	11.98 (0.12)				

gDW: gram dry weight, LEH: length of extraradical AMF hyphae, AMF: arbuscular mycorrhizal fungi, RLC: root length colonized.

### Phase 1

#### Soil biota community effects

Root biomass of the plant community was 20% higher when grown with SEG 37 than with SEG 33, while total shoot and plant biomass as well as plant diversity, measured as Shannon's H′ on the basis of shoot biomass was not affected by the soil biota community (Tables 1 and 2). However, the soil biota community affected shoot biomass of three out of six plant species, with *A. millefolium* growing 83% larger with soil biota community SEG 33 than with SEG 37, while *T. officinale* and *H. lanatus* grew 40% and 28% larger with SEG 37 than with SEG 33.

**Table 2:** Effects of soil biota community (SB, SEG 33 and SEG 37) and *Agriotes* presence (AP1, without and with) on plant and AMF parameters and soil CN in phase 1.

	SB		AP1		SB x AP1	
	df:1		df:1		df:1	
	F	p	F	p	F	p
Shannon's H	0.28	ns	0.75	ns	0.02	ns
Plant biomass total	3.10	ns	0.01	ns	0.00	ns
Root biomass total	13.00	***	1.98	ns	1.46	ns
Shoot biomass total	0.01	ns	0.61	ns	0.55	ns
Shoot biomass						
*A. millefolium*	30.65	***	9.54	**	7.11	*
*P. lanceolata*	0.16	ns	9.94	**	2.57	ns
*T. officinale*	4.21	*	0.16	ns	0.82	ns
*H. lanatus*	9.66	**	0.57	ns	0.18	ns
*P. pratensis*	0.35	ns	1.50	ns	2.52	ns
*T. repens*	0.62	ns	3.10	ns	1.02	ns
LEH	22.65	***	1.68	ns	0.62	ns
AMF community in soil	14.36	**	0.16	ns	2.42	ns
No of AMF species in soil	5.77	*	0.47	ns	0.74	ns
Total AMF *(P. lanceolata)*	4.15	*	0.01	ns	0.10	ns
Arbuscules *(P. lanceolata)*	2.84	ns	0.00	ns	0.11	ns
Soil CN ratio	0.62	ns	0.01	ns	0.73	ns

significance level:

* p<0.05,

** p<0.01,

*** p<0.001,

ns = not significant, LEH: length of extraradical AMF hyphae, AMF: arbuscular mycorrhizal fungi.

The two soil biota communities differed in their AMF community composition in soil, with 1.4 AMF species more present and extraradical hyphae being 2.6 times longer in soil of SEG 37 than of SEG 33. Total AMF structures of SEG 37 colonized 27% more of *P. lanceolata* roots length than AMF of SEG 33. Soil CN ratio did not differ for the two soil biota communities.

#### Root herbivory effects

**Figure 1:**
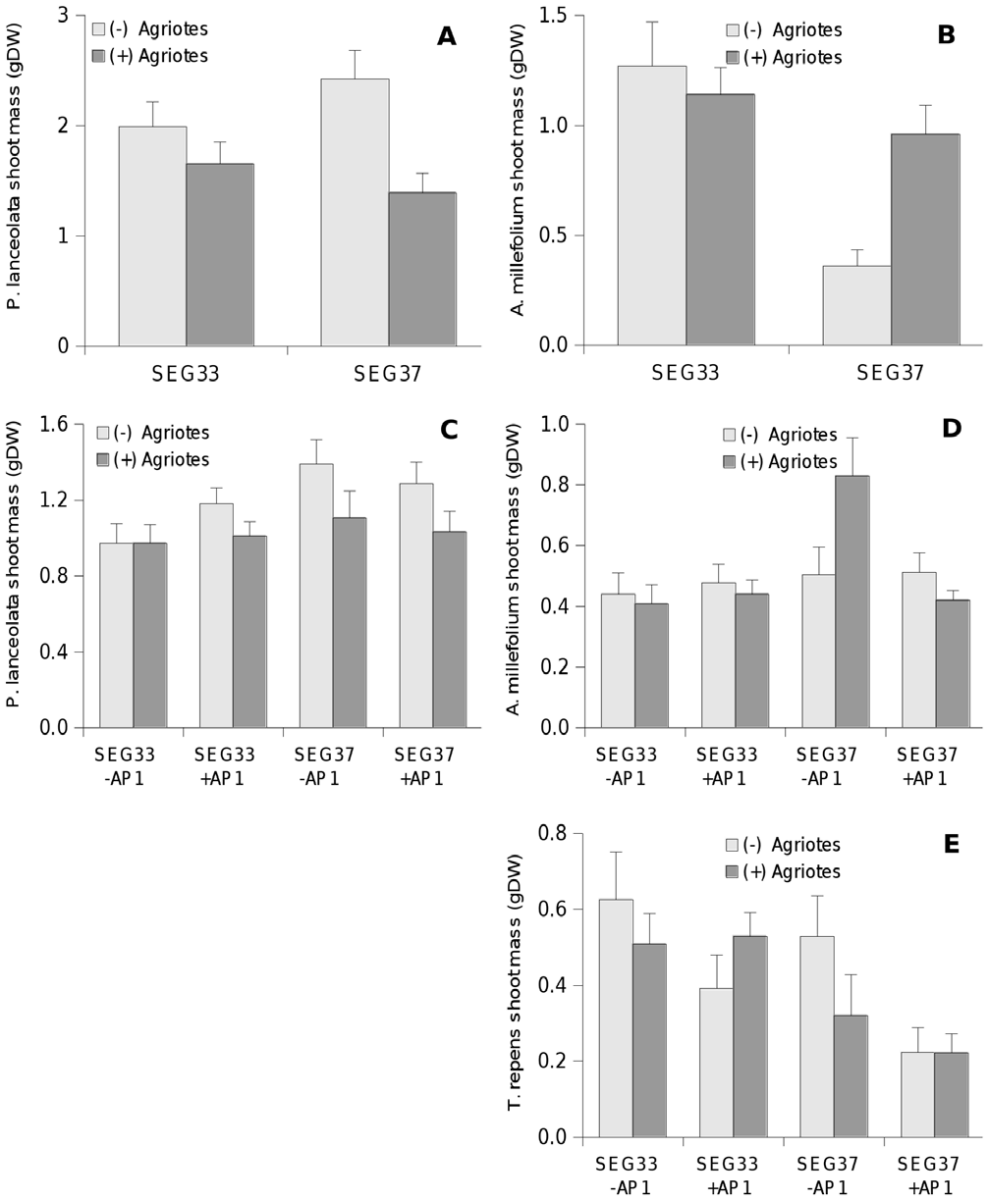
Shoot biomass (mean + se) of plants affected by the presence of *Agriotes* larvae. Phase 1 (A) *P. lanceolata* (B) *A. millefolium*; phase 2 (C) *P. lanceolata* (D) *A. millefolium*, (E) *T repens;* SEG33, SEG37: soil biota from respective grassland sites; −AP1, +AP1: untrained and *Agriotes* trained soil from phase 1, respectively.

Plant community parameters did not respond to the presence of *Agriotes* larvae. On the level of single plants, *Agriotes* larvae reduced shoot biomass of *P. lanceolata* (Figure 1A) by 31% on average and the effect did not differ significantly for the two soil biota communities. On the contrary, shoot biomass of *A. millefolium* (Figure 1B) was 2.7 times higher in the presence than in the absence of *Agriotes* larvae, but only when grown in soil biota community SEG 37. AMF parameters as well as the soil CN ratio were not affected by the presence of *Agriotes* larvae.

### Phase 2

#### Soil biota community effects

Soil biota community effects on plant community biomasses were similar to phase 1, with root biomass of the plant community being 14% higher when grown with SEG 37 than with SEG 33, while total shoot and plant biomass was not affected (Tables 1 and 3). The plant community was 9% more diverse when grown with SEG 33 than with SEG 37. The effect of the soil biota community on single plant species differed from that in phase 1, with *P. pratensis* and *T. repens* now being affected and growing 400% and 61%, larger with soil biota community SEG 33 than SEG 37, respectively, while responsive plant species in phase 1 (*A. millefolium*, *T. officinale*, *H. lanatus*) were not affected anymore.

**Table 3:** Effects of soil biota community (SB, SEG 33 and SEG 37), *Agriotes* training (AP1, *Agriotes* presence in phase 1, untrained and trained) and *Agriotes* presence (AP2, without and with) on plant and AMF parameters in phase 2.

	SB		AP1		AP2		SB X AP1		SB x AP2		AP1 x AP2		SB x AP1 x AP2	
	df:1		df:1		df:1		df:1		df:1		df:1		df:1	
	F	p	F	p	F	p	F	p	F	p	F	p	F	p
Shannon's H	22.17	***	0.04	ns	2.30	ns	0.44	ns	0.04	ns	0.44	ns	0.02	ns
Plant biomass total	1.90	ns	5.26	*	0.31	ns	0.20	ns	1.03	ns	2.11	ns	0.59	ns
Root biomass total	4.42	*	3.14	ns	0.01	ns	0.34	ns	0.46	ns	2.58	ns	1.06	ns
Shoot biomass total	0.02	ns	3.98	*0.05*	0.84	ns	0.02	ns	0.45	ns	0.04	ns	0.02	ns
Shoot biomass														
*A. millefolium*	2.31	ns	2.26	ns	0.02	ns	4.76	*	0.42	ns	3.77	*0.06*	3.66	*0.06*
*P. lanceolata*	3.64	ns	0.05	ns	7.81	**	1.40	ns	2.14	ns	0.35	ns	0.64	ns
*T. officinale*	3.15	ns	5.32	*	0.92	ns	3.39	ns	0.12	ns	1.01	ns	0.04	ns
*H. lanatus*	2.90	ns	3.24	ns	3.62	ns	0.25	ns	1.17	ns	0.16	ns	0.54	ns
*P. pratensis*	28.51	***	0.48	ns	1.74	ns	0.13	ns	0.01	ns	0.00	ns	0.13	ns
*T. repens*	8.14	**	4.51	*	0.10	ns	0.43	ns	2.21	ns	4.98	*	0.06	ns
LEH	0.00	ns	0.97	ns	0.02	ns	7.12	*	4.89	*	0.67	ns	0.00	ns
AMF community in soil	7.93	**	0.99	ns	−0.39	ns	4.99	**	3.44	*	2.57	ns	0.37	ns
No of AMF species in soil	0.41	ns	0.00	ns	0.04	ns	6.11	*	3.57	ns	0.44	ns	0.03	ns
AMF community in roots	9.30	***	0.06	ns	−0.01	ns	0.26	ns	1.93	ns	0.51	ns	0.11	ns
No of AMF species in roots	9.10	**	0.49	ns	0.74	ns	0.19	ns	0.03	ns	0.47	ns	0.42	ns
Total AMF *(P. lanceolata)*	15.21	**	4.98	*	0.22	ns	1.20	ns	0.09	ns	6.95	*	4.65	*
Arbuscules *(P. lanceolata)*	4.91	*	0.05	ns	0.03	ns	0.26	ns	5.72	*	6.40	*	0.17	ns

significance level:

* p<0.05,

** p<0.01,

*** p<0.001,

ns = not significant, LEH: length of extraradical AMF hyphae, AMF: arbuscular mycorrhizal fungi.

The two soil biota communities differed in their AMF community composition in soil and *P. lanceolata* roots, with 1.09 AMF species more present in *P. lanceolata* roots grown in SEG 37 than in SEG 33. The length of extraradical hyphae did not differ for AMF from the two soil biota communities. Contrary to phase 1, AMF from SEG 33 colonized a higher percentage of *P. lanceolata* roots length than AMF from SEG 37. This was true for mycorrhizal structures in total as well as for arbuscules (30% and 45%, higher colonization with SEG 33 than SEG 37, respectively).

#### Root herbivore history effects

Total plant biomass was higher (9%; Table 1) when grown in *Agriotes* trained soil (presence of *Agriotes* larvae in phase 1) compared to untrained soil. The same tendency was observed for total shoot biomass (7%; lme: p = 0.05). Root biomass and plant diversity were not affected by the herbivore history of the soil. On the level of single plants, *Agriotes* trained soil facilitated shoot growth of *T. officinale* by 32% but reduced shoot growth of *T. repens* by 31% compared to untrained soil.

**Figure 2:**
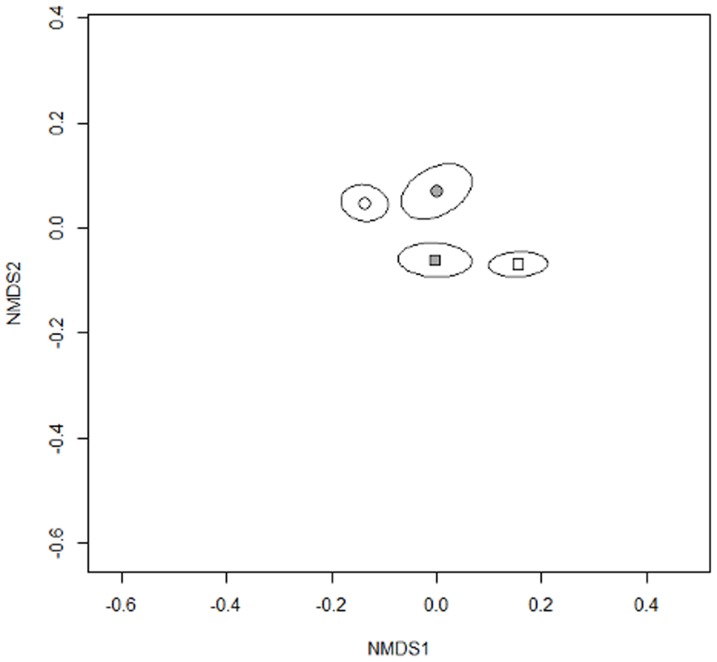
Non-metric multidimensional scaling plot of AMF communities in soils after phase 2. Circles and squares give centroids (means) of AMF communities for SEG33 and SEG37, respectively, open symbols show those of untrained and filled symbols those of trained AMF communities. Ellipsoids give standard error. The two soil biota origins are clearly differentiated along the second axis while the *Agriotes* training leads to opposing community shifts along the first axis.

The effect of the *Agriotes* training on AMF community composition differed for the two soil biota communities (Figure 2). The community of *Agriotes* trained AMF consisted of 0.93 more species and 44% shorter extraradical hyphae for SEG 33 but of 0.42 fewer species and 34% longer hyphae for SEG 37 compared to the respective untrained AMF communities. Total structures of *Agriotes* trained AMF colonized 14% less of *P. lanceolata* roots length than structures of untrained AMF. Other AMF parameters were not affected by the *Agriotes* training.

#### Root herbivory effects

As in phase 1, plant diversity as well as plant community biomasses did not respond to the presence of *Agriotes* larvae. Also in accordance with phase 1, *Agriotes* larvae reduced shoot biomass of *P. lanceolata* (Figure 1C) by 15% on average and statistically independent of the soil biota community and *Agriotes* training, while shoot biomass of *A. millefolium* (Figure 1D) was not affected by the presence of *Agriotes* larvae when grown in soil biota community SEG 33, but enhanced by 65% when grown in untrained SEG 37. As indicated by a marginally non-significant three way interaction (lme: p = 0.06) of original soil biota community (SB), *Agriotes* training (AP1) and *Agriotes* presence (AP2), the positive effect of the presence of *Agriotes* larvae with soil biota community SEG 37 was lost when the soil was already *Agriotes* trained. Shoot biomass of *T. repens* (Figure 1E) was reduced by 28% by *Agriotes* larvae in phase 2, but only with untrained soil.

Contrarily to phase 1, AMF parameters were affected by the presence of *Agriotes* larvae. For the community composition in soil, the length of extraradical hyphae (LEH) and the percentage of *P. lanceolata* root length colonized by arbusclues the effect depended on the original soil biota community. LEH from SEG 33 was reduced by 27% (from 1.9 (se = 0.3) to 1.4 (se = 0.4) m/g DW soil) by *Agriotes* larvae while LEH from SEG 37 was increased by 49% (from 1.3 (se = 0.3) to 2.0 (se = 0.3) m/g DW soil). For AMF arbuscules an additional interaction of the factors *Agriotes* training and presence of *Agriotes* larvae resulted in colonization by *Agriotes* trained AMF being less negatively affected in SEG 33 and positively affected in SEG 37 by the presence of *Agriotes* larvae compared to arbuscules colonization by untrained AMF (50% reduction (from 22.0 (se = 2.7) to 11.0 (se = 1.0) %RLC) in untrained SEG 33 compared to no effect in trained SEG 33 (mean 17.1 (se = 4.4) %RLC), and 210% increase (from 5.3 (se = 0.5) to 16.3 (se = 1.5) %RLC) in trained SEG 37 compared to no effect in untrained SEG 37 (mean 12.5 (se = 2.4) %RLC)). On the level of total AMF structures, the same two way interaction of the presence of *Agriotes* larvae and *Agriotes* training as well as a three way interaction including original soil biota led to a similar effect for total AMF structures from SEG 37 (38% increase (from 39.0 (se = 3.4) to 54.0 (se = 2.4) %RLC) in trained SEG 37), whereas total AMF structures from SEG 33 were not affected (mean 69.2 (se = 4.9) %RLC).

## Discussion

The study aimed at determining root herbivore history effects of *Agriotes* spp. larvae on a grassland plant community. As hypothesized, the root herbivore history of the soil influenced plant growth as well as plant growth response to new root herbivore attack. It affected plant community productivity (but not composition), showing for the first time that root herbivore induced changes in soil conditions can impact plant communities in a subsequent season. The root herbivore history of the soil proved to be a stronger driver of plant growth on the community level than an actual root herbivore attack which did not affect plant community parameters.

The two phases of the experiment differed in plant growth and community composition due to different growth conditions. Pots in phase 2 supported less plant biomass than pots in phase 1, presumably because they contained a smaller proportion of steamed, nutrient rich background soil.

### Soil biota community effects

The original soil biota community affected the root biomass of the plant community and the shoot growth of several plant species in both phases of the experiment. The significant effects of the soil biota treatment confirm that (i) the two soil biota communities differed in their abundance/composition and (ii) the structure of soil biota communities can have pronounced impact on plant community structure [37].

Differences in community composition were also directly confirmed for AMF. Similar CN ratios of soil biota inocula (SEG 33 = 11.0 , SEG 37 = 11.8) suggest that factors other than nutrient provision by the decomposer community, like plant species specific interactions with beneficial or detrimental soil organisms were involved in creating the soil biota effect. The identity of the plant species that were affected by the soil biota community differed between the two phases of the experiment. Additionally, plant diversity was only affected by the soil biota community in phase 2. Differences in soil biota effects between phase 1 and 2 may be ascribed to differences in (i) soil biota/AMF community composition due to plant species specific accumulation of associated organisms (plant-soil feedback effects [38]) during phase 1 and due to the dry dormancy between phases and/or (ii) nutrient content of the growth substrate, with effects of soil organisms being more pronounced under the nutrient poor conditions [39] in phase 2.

### Root herbivore history effects

The *Agriotes* training of the soil enhanced plant community productivity (total shoot and plant growth). Effects of the *Agriotes* training indicate changes in plant growth conditions due to the root herbivore that persisted even when the root herbivore was no longer present [7]. Our results show that these root herbivore history effects are even detectable at the plant community level. On the level of single plants *Agriotes* training enhanced shoot growth of *T. officinale* but reduced shoot growth of *T. repens*.

Effects on plant community biomasses and the N indicator species *T. officinale* [40] were positive, pointing to enhanced nutrient supply. Similar CN ratios of untrained and trained soil inocula suggest that nutrient provision by the decomposer community did again not cause the training effect. Instead, AMF communities differed between trained and untrained soils. The difference was not preceded by an actual root herbivore effect on AMF communities in the first phase of the experiment, indicating that it may have been generated through an influence on viability of AMF spores that changed community structure only after the dry dormancy between the two phases of the experiment. Though AMF community shifts due to root herbivore training differed depending on the original soil biota community, they may still have been towards higher abundance of species beneficial for those plants that contributed to enhanced plant community productivity (e.g. *T. officinale*) in both soil biota communities. The reduction in *T. repens* shoot biomass points to reduced competitive advantage through its symbiotic N fixation at improved nutrient acquisition by AMF for other plants.

### Root herbivory effects

Plant community parameters did not respond to the presence of the root herbivore. For single plant species our results show that actual root herbivory effects are influenced by the root herbivore history of the soil, indicating that root herbivore induced changes in plant growth conditions also determine plant responses to new root herbivore attack.

Shoot growth of *A. millefolium* was facilitated by *Agriotes* larvae when grown in untrained soil biota community SEG 37, however, this effect was lost when the soil was already *Agriotes* trained. Shoot biomass of *T. repens* was reduced by *Agriotes* larvae, but only with the two untrained soils in phase 2. AMF communities in phase 2 were affected by the herbivore presence (in interaction with the original soil biota community). However, the actual root herbivore effect on AMF was less strong than the herbivore history effect. When grown individually in SEG 37 soil [21] shoot biomass of *A. millefolium* and *T. repens* did not respond to herbivory by *Agriotes* larvae. Thus, in the present study, responses of *A. millefolium* and *T. repens* to root herbivory may be best explained by (i) herbivore history induced differences in AMF species composition and (ii) shifts in the plant community composition. In untrained SEG 37 soil, roots of *A. millefolium* may have replaced those of other members of the community that were consumed by the root herbivore. Low root losses that did not result in changes in shoot biomass may have been sufficient, as *A. millefolium* responds strongly to release from below ground competition [41]. The shift was not detected in *Agriotes* trained SEG 37 where plants were potentially better nourished by AMF (see *Root herbivore history effects*) and thus able to compensate the herbivore damage [2], and in SEG 33 where plant community root biomass was lower and release from below ground competition thus less pronounced. *Trifolium repens* did not respond to *Agriotes* presence in phase 1 when it was the strongest competitor in the plant community but in phase 2 where it was less strong. In phase 2 the shoot mass reduction only occurred with untrained, potentially less well nourished plants where *T. repens* presumably benefited more from its N fixing symbiosis, thus, suggesting a root herbivore effect on the symbiosis [42] that became apparent under these conditions. In contrast, shoot biomass of *P. lanceolata* was reduced by *Agriotes* larvae in both phases of the experiment and the effect did not differ significantly for the two original soil biota communities and the training treatments, suggesting independence of soil conditions for the root herbivore effect on this plant species. This was despite the fact that the AMF community in *P. lanceolata* roots differed significantly between the two original soil biota treatments in phase 2 and quantitative root colonization parameters were additionally affected by the herbivore history and herbivore presence. In accordance, this plant species showed similar growth response to several AMF species and no connection of growth response to the degree of root colonization in other studies ([43] and [44], resp.). Substantial root biomass loss in the presence of *Agriotes* larvae in two experiments where *P. lanceolata* was grown individually ([19], [21]) point to a strong direct grazing effect that may have overridden indirect root herbivore effects for this plant species.

In conclusion, our study found evidence for root herbivore history effects on grassland plant communities. The effects may partly be explained by herbivore induced shifts in the AMF community. Interestingly, the root herbivore history of the soil proved to be a stronger driver of plant growth on the community level than an actual herbivore attack. History effects have to be taken into account when predicting the impact of root herbivores in grasslands.

## Supporting Information

Tables S1 **Includes the tables S1 and S2.** Plant biomass data and AMF parameters in different soil treatments in phase1 and phase2.
(DOCX)

Protocol S1 **Detailed protocol for characterization of AMF community.**
(DOC)
