# Supplementary material for: The Root Herbivore History of the Soil Affects the Productivity of a Grassland Plant Community and Determines Plant Response to New Root Herbivore Attack
Source: PLoS One. 2013 Feb 18;8(2):e56524. doi: 10.1371/journal.pone.0056524 (PMC3575479; doi:10.1371/journal.pone.0056524)
Supplement: Tables S1 — Includes the tables S1 and S2. Plant biomass data and AMF parameters in different soil treatments in phase1 and phase2. (DOCX) [file pone.0056524.s001.docx]

**Supporting table S1. Plant biomass data (means (se) in different soil treatments (soil biota communities SGE 33 and SEG 37, *Agriotes* absence (-) or presence (+)) in phase1 (P1)/phase2 (P2).**

|  |  |  | Shoots [gDW/plant] | | | | | |
| --- | --- | --- | --- | --- | --- | --- | --- | --- |
| Soil biota community | Agriotes treatment P1/P2 | Roots total [gDW/pot] | *A.millefolium* | *P. lanceolata* | *T. officinale* | *H. lanatus* | *P. pratensis* | *T. repens* |
| Phase 1 | | |  |  |  |  |  |  |
| SEG 33 | - | 7.76 (0.40) | 1.27 (0.20) | 1.99 (0.23) | 0.22 (0.03) | 3.06 (0.25) | 0.22 (0.03) | 6.23 (0.68) |
| SEG 33 | + | 7.68 (0.47) | 1.14 (0.12) | 1.65 (0.20) | 0.28 (0.06) | 2.97 (0.24) | 0.23 (0.04) | 6.75 (0.82) |
| SEG 37 | - | 9.77 (0.33) | 0.36 (0.07) | 2.42 (0.26) | 0.38 (0.08) | 4.01 (0.37) | 0.30 (0.04) | 4.97 (0.67) |
| SEG 37 | + | 8.68 (0.45) | 0.96 (0.13) | 1.39 (0,18) | 0.32 (0.06) | 3.69 (0.20) | 0.20 (0.04) | 6.91 (0.63) |
| Phase 2 | | |  |  |  |  |  |  |
| SEG 33 | - / - | 2.08 (0.20) | 0.44 (0.07) | 0.97 (0.10) | 0.78 (0.13) | 0.81 (0.14) | 0.07 (0.01) | 0.63 (0.12) |
| SEG 33 | - / + | 1.93 (0.14) | 0.41 (0.06) | 0.97 (0.10) | 0.73 (0.10) | 1.00 (0.15) | 0.07 (0.02) | 0.51 (0.08) |
| SEG 33 | + / - | 2.34 (0.12) | 0.48 (0.07) | 1.18 (0.08) | 0.73 (0.11) | 1.09 (0.19) | 0.08 (0.01) | 0.39 (0.09) |
| SEG 33 | + / + | 2.32 (0.18) | 0.44 (0.05) | 1.01 (0.08) | 0.88 (0.13) | 1.07 (0.18) | 0.09 (0.02) | 0.53 (0.06) |
| SEG 37 | - / - | 2.49 (0.28) | 0.50 (0.09) | 1.40 (0.13) | 0.73 (0.12) | 0.50 (0.15) | 0.01 (0.01) | 0.53 (0.11) |
| SEG 37 | - / + | 2.27 (0.13) | 0.83 (0.13) | 1.11 (0.14) | 0.78 (0.15) | 0.76 (0.14) | 0.02 (0.01) | 0.32 (0.11) |
| SEG 37 | + / - | 2.35 (0.17) | 0.51 (0.06) | 1.29 (0.12) | 1.11 (0.13) | 0.77 (0.20) | 0.02 (0.01) | 0.22 (0.07) |
| SEG 37 | + / + | 2.74 (0.16) | 0.42 (0.03) | 1.03 (0.11) | 1.28 (0.16) | 1.09 (0.14) | 0.02 (0.01) | 0.22 (0.05) |

gDW: gram dry weight

**Supporting table S2. AMF parameters in different soil treatments (soil biota communities SGE 33 and SEG 37, *Agriotes* absence (-) or presence (+)) in phase1 (P1)/phase2 (P2).**

| Soil biota community | Agriotes treatment P1/P2 | LEH | Arbuscules | AMF total | AMF species  in soil | AMF species in roots |
| --- | --- | --- | --- | --- | --- | --- |
|  |  | [m/gDW soil] | [% *P. lanceolata* RLC] | | [no] | [no] |
| Phase 1 | | |  |  |  |  |
| SEG 33 | - | 0.40 (0.05) | 7.8 (1.6) | 19.2 (2.1) | 3.3 (0.6) |  |
| SEG 33 | + | 0.54 (0.05) | 8.6 (2.1) | 18.7 (2.7) | 4.2 (0.6) |  |
| SEG 37 | - | 1.29 (0.15) | 5.8 (0.9) | 23.5 (2.7) | 5.2 (0.6) |  |
| SEG 37 | + | 1.18 (0.16) | 5.6 (1.1) | 24.6 (2.5) | 5.1 (0.5) |  |
| Phase 2 | | |  |  |  |  |
| SEG 33 | - / - | 2.43 (0.32) | 22.0 (3.1) | 73.4 (3.8) | 2.1 (0.3) | 3.1 (0.4) |
| SEG 33 | - / + | 1.72 (0.58) | 11.0 (5.4) | 69.6 (1.4) | 2.7 (0.2) | 2.9 (0.5) |
| SEG 33 | + / - | 1.32 (0.37) | 16.2 (4.0) | 67.0 (3.4) | 2.9 (0.5) | 3.0 (0.6) |
| SEG 33 | + / + | 1.01 (0.21) | 18.0 (7.0) | 66.6 (5.3) | 3.6 (0.5) | 2.8 (0.2) |
| SEG 37 | - / - | 1.18 (0.23) | 11.6 (3.6) | 66.2 (2.4) | 3.8 (0.5) | 4.6 (0.5) |
| SEG 37 | - / + | 1.61 (0.24) | 13.4 (4.8) | 53.4 (2.3) | 3.2 (0.5) | 3.9 (0.4) |
| SEG 37 | + / - | 1.43 (0.45) | 05.3 (5.4) | 39.0 (0.9) | 2.7 (0.5) | 3.8 (0.4) |
| SEG 37 | + / + | 2.32 (0.43) | 16.3 (3.7) | 54.0 (2.3) | 2.6 (0.6) | 3.9 (0.6) |

gDW: gram dry weight, LEH: length of extraradical AMF hyphae, AMF: arbuscular mycorrhizal fungi, RLC: root length colonized
