# Supplementary material for: The Root Herbivore History of the Soil Affects the Productivity of a Grassland Plant Community and Determines Plant Response to New Root Herbivore Attack
Source: PLoS One. 2013 Feb 18;8(2):e56524. doi: 10.1371/journal.pone.0056524 (PMC3575479; doi:10.1371/journal.pone.0056524)
Supplement: Protocol S1 — Detailed protocol for characterization of AMF community. (DOC) [file pone.0056524.s002.doc]

*Detailed protocol for characterization of AMF community*

DNA was extracted from substrate and roots using the PowerSoil® DNA Isolation Kit (MO BIO Laboratories, Inc., Carlsbad, Canada). Roots were heated to 60 °C for 10 min prior to extraction. The obtained DNA was used to amplify a widely used gene region within the ribosomal small subunit (SSU). For root samples we used a nested PCR with the general fungal primer pair NS1/NS4 [1] as outer primers and the AMF specific primer pair NS31/AML2 ([1] – [2]) as inner primers. For soil samples, only the inner primers were used. In order to create a experiment wide database of AMF OUT diversity, we pooled PCR products from roots of both phases and soil from phase 1. The former should cover AMF OTUs actively colonizing the roots, whereas the latter may also include OTUs resting in the soil. The root and soil pools were separately cloned into the pGEM®-T easy vector and transformed into chemically competent Escherichia coli (Promega GmbH, Mannheim, Germany). Positive clones were identified by blue/white screening and the presence of SSU inserts was confirmed using vector-targeting primers M13F and M13R. From the root and soil pool, 53 and 76 clones were sequenced by LGC Genomics (Berlin, Germany), respectively. Sequences were then grouped into 81 Glomeromycota and 48 non-Glomeromycota (i.e. unspecific amplicons) according to the results obtained by BLASTN search in GenBank [3]. Glomeromycota sequences were aligned using MAFT version 6 (http://align.bmr.kyushu-u.ac.jp/mafft/online/server/). The phylogenetic relations were inferred applying the Kimura-2-parameter model with the Neighbor Joining (NJ) algorithm as implemented in MEGA4 [4]. The confidence of branching was assessed by computing 1000 bootstrap resamplings. Sequences falling into one clade with bootstrap support of at least 80% and showing sequence identities of at least 98% were considered as an OTU and numbered consecutively.

This analysis resulted in eight OTUs and a rarefaction analysis (analytical rarefaction version 1.3 software; S. Holland; <http://www.uga.edu/strata/software/Software.html>) indicated that these cover ~90% of the AMF diversity present in the pools. Extrapolation of a hyperbolic function fit on these data showed that at least 10 x more sequences would have been necessary to detect all AMF OTUs.

Using the REPK online software [5] we found the three restriction enzymes DpnII, Hin6I and TaqI sufficiently discriminating between the OTUs found in the sequence library. PCR reactions were redone under the same conditions as described above with the only exception of a fluorescent label (6-FAM) attached to the forward primer NS31. 80 ng of each PCR product were digested individually with each enzyme for 2 hours at 37 °C or 65 °C (for TaqI). Fragment lengths were determined at the SMB Service GmbH (Berlin, Germany) using a customized ROX size standard on a ABI 3100 capillary sequencer (Applied Biosystems Carlsbad, CA, USA). Obtained data were processed using GeneMapper software version 3.7 (Applied Biosystems, 2004). The R (R Development Core Team, 2010) package, TRAMPR [6], was used for matching TRFLP profiles with the database.

References

1. Simon L, Lalonde M, Bruns TD (1992) Specific amplification of 18s fungal ribosomal genes from vesicular-arbuscular endomycorrhizal fungi colonizing roots. Applied and Environmental Microbiology 58: 291-295.
2. Lee J, Lee S, Young JPW (2008) Improved PCR primers for the detection and identification of arbuscular mycorrhizal fungi. Fems Microbiology Ecology 65: 339-349.
3. Altschul SF, Madden TL, Schaffer AA, Zhang JH, Zhang Z, et al. (1997) Gapped BLAST and PSI-BLAST: a new generation of protein database search programs. Nucleic Acids Research 25: 3389-3402.
4. Tamura K, Dudley J, Nei M, Kumar S (2007) MEGA4: Molecular evolutionary genetics analysis (MEGA) software version 4.0. Molecular Biology and Evolution 24: 1596-1599.
5. Collins RE, Rocap G (2007) REPK: an analytical web server to select retriction endonucleases for terminal restriction fragment length polymorphism analysis. Nucleic Acids Research 35: W58-W62.
6. FitzJohn RG, Dickie IA (2007) TRAMPR: An R package for analysis and matching of terminal-restriction fragment length polymorphism (TRFLP) profiles. Molecular Ecology Notes 7: 583-587.
